# Supplementary material for: Are carbon emissions trading and green financial instruments synergistic? -Comprehensive quantitative research based on content analysis
Source: PLoS One. 2024 Mar 7;19(3):e0298601. doi: 10.1371/journal.pone.0298601 (PMC10919601; doi:10.1371/journal.pone.0298601)
Supplement: S1 Data — (ZIP) [file pone.0298601.s001.zip › Data/地级市Z_F工作报告.zip!/╣π╢1⁄2/╔╪╣╪╩╨/└┤╫╘╣1⁄2╓┌║┼í╛┬φ┐╦╩2╛▌═°í┐_2012.docx]

各位代表：

我代表市十二届人民政府，向大会作工作报告，请予审议，并请市政协委员和其他列席人员提出意见。

本届政府工作回顾

2007至2011年，市政府在省委、省政府和市委的正确领导下，在市人大及其常委会和市政协的监督支持下，深入学习实践科学发展观，团结带领全市人民，着力加快转变经济发展方式，有效应对国际金融危机冲击，战胜严重自然灾害，较好地完成了“十一五”规划和十二届人大各次会议确定的主要目标任务，“十二五”规划开局良好，各项建设取得了新成就。

一、着力提高经济发展质量，改善民生成效显著

2011年，全市实现生产总值820亿元、比上年增长12%（预计数，下同），其中第一产业增加值比上年增长6%，第二产业增长14.4%，第三产业增长11%。三次产业比例由2006年的14.4：46：39.6调整为2011年的13.8：42.7：43.5。人均生产总值2.8万元、比上年增长11.5%，五年年均增长13.4%。民营经济增加值年均增长12.7%。县域生产总值年均增长15.9%，占全市比重比2006年提高6个百分点、达49%。全市地方财政一般预算收入53.9亿元、增长18.9%，五年增长1倍。金融机构存贷款余额五年年均分别增长13.1%和18.8%。各类市场主体突破10万户，五年新增3万多户。

2011年，城镇居民人均可支配收入16085元，农村居民人均纯收入7260元，分别比上年增长12.8%和14.9%。市区居民消费价格指数涨幅低于全国全省水平。全市财政民生支出比重超过60%，比2006年提高6.6个百分点。五年来，我市《妇女儿童发展规划（2001—2010）》目标任务全面完成；市政府承诺办好的44件实事基本完成；基本建立公共就业服务体系，城镇新增就业26.8万人，城镇失业人员再就业18.9万人。2011年城镇登记失业率为2.62%，“零就业家庭”户数实现动态归零。实行新农合、新农保、城居保等社会保障制度，基本建立覆盖城乡各种从业人员、惠及各类群体的养老保险和医疗保险体系。企业退休人员人均养老金比2006年翻一番。社会救助、慈善公益、残疾人事业和老龄事业加快发展。五年来，共救助受灾群众211.1万人次，帮助灾民重建住房8511套；54.6万人次享受低保待遇，发放医疗救助金救助13.4万人次，2.8万名贫困家庭学生免费接受高中阶段教育；办理法律援助案件6035件，建成保障性住房5581套，完成了省政府下达的保障性住房开工建设任务。启动了原曲仁矿棚户区改造一期工程。扶贫开发“双到”工作实现三年任务两年基本完成。3.3万户贫困户、12.9万贫困人口人均纯收入达到2500元以上的脱贫标准，分别占总数的87.9%和89.6%，352个贫困村集体经济收入达到3万元以上。贫困地区生产生活条件明显改善，“造血”功能显著增强。

二、扎实推进“双转移”，现代产业体系框架初步形成

2008至2011年，全市累计承接产业转移项目1507个，到位资金360亿元、年均增长17%；省级产业转移工业园规模以上工业增加值年均增长34%，占全市规模以上工业比重由6.8%提高到15%，装备制造、精细化工等主导产业集聚发展。2011年全市完成农村劳动力技能培训3.1万人，转移就业7.5万人。2008至2010年我市“双转移”工作连续三年被评为全省“双优”。2011年我市成为广东省优先扶持的承接产业转移重点区域之一，莞韶产业转移工业园被列为省承接产业转移重点扶持园区。南雄精细化工基地成功申报省级产业转移工业园，成为我省欠发达地区县（市）探索产业集聚的一张“名片”。2011年全市吸收外商直接投资、进出口总额分别比上年增长12%和11.1%，首次进入中国外贸100强城市行列。2010年以来，全市共有26个项目列入省现代产业500强项目。

2011年全市工业增加值300亿元，比上年增长13.7%。五年来，高新技术产品数量增长1倍、产值增长1.5倍，新建66.9万千瓦发电装机容量，新增143.5万吨钢材等一批重要生产能力。启动省市共建装备基础零部件先进制造业基地。五年实施科技项目739项，专利申请量、授权量五年分别增长1.2倍和1.7倍，新增注册商标3751件。连续两次通过国家科技进步考核。建成现代化标准农田36万亩以及大批蔬菜、水果、生猪、花卉、茶叶、油茶、蚕桑、烟叶、中药材生产基地，建立粤北现代农业示范园区和粤台（韶关）农业合作试验区，农业综合生产能力逐年提高，粮食安全责任制通过省考核。2011年农业龙头企业、农民专业合作社带动农户26万户，户均增收6000多元。全市无公害农产品、绿色食品、有机食品达304个，地理标志保护产品12个，其中五年分别新增257个和11个。农业机耕率达81%，农机总动力达140万千瓦。五年引进农业项目200多个，实际到位资金20多亿元。2011年全市社会消费品零售总额比上年增长16.8%，商品房销售额增长10%。接待旅游者人次、旅游收入分别增长15%和20%，旅游业快速发展。丹霞山成功申报成为世界自然遗产，旅游经济指标翻倍增长。五年新增省旅游强县（市）3个、国家级4A旅游景区4个，新增旅店床位1.3万张、星级酒店18家，按五星级标准建成酒店6家。

三、不断扩大投资规模，基础设施建设明显加强

2011年全市完成固定资产投资471亿元、增长16%，五年累计完成1762亿元。其中重点项目完成投资850亿元，韶关电网改造、500千伏坪B线路、坪B电厂三期扩建等一批电力产业项目建成投产，韶钢节能减排、丹冶扩建等项目顺利推进。五年交通项目建设完成投资331亿元，新建高速公路127公里、一级公路76.7公里。武广高速铁路（韶关段）、韶赣高速公路建成通车。赣韶铁路（粤境段）、广乐高速公路（韶关段）、北江航道整治和港口建设进展顺利。北江航道（韶关段）等级提升至五级，可昼夜通航300吨级船舶。西气东输二线韶关段建成，省天然气管网一期工程韶关支干线基本建成。湾头水利枢纽建成运行，乐昌峡水利枢纽主体工程完工并具备防洪功能。全市兴建防洪堤232.7公里，中小型水库除险加固405宗。城乡防灾减灾能力显著提高，韶关市区防洪标准由20年一遇提高到100年一遇。

四、统筹加快城乡建设，粤北区域中心城市初具规模

2011年末韶关市区建成区面积81.8平方公里、比2006年增加3.5平方公里，市区常住人口达100万人。五年新增城市主干道53.3公里。武广高铁韶关站及站前广场、韶关大道、帽峰大桥和韶南大道改造等一批项目建成投入使用，芙蓉大道、百年东街等69个项目动工建设。五年来，完成近4000公里农村公路硬化，建成800间农家书屋和1112个行政村农民体育健身工程，帮助6.3万户农民改建住房，解决52.7万人饮水安全问题，新建沼气池8.5万个。全市90%以上的乡镇实施了乡村“清洁美”工程。

全国生态文明建设试点市工作扎实推进。五年造林213.4万亩。2008年雨雪冰冻灾害中受损的森林基本修复。2011年全市森林面积、森林覆盖率、活立木蓄积量、自然保护区和珍稀动植物数量面积均居全省前列，华南虎繁育取得可喜成果。土地矿产资源保护力度加大，五年新增耕地16万亩，连续10年实现耕地占补平衡。全市单位生产总值能耗比2006年下降19.2%，城镇生活污水集中处理率达77.6%，全面完成节能减排目标任务。

五、积极构建和谐社会，各项事业不断发展

社会主义思想道德建设和精神文明创建活动不断加强，公民文明素质和城乡文明程度逐步提高，一批“韶关好人”荣登“中国好人”榜。深入开展双拥活动和国防教育，国防后备力量建设进一步加强。完成“五五”普法，启动“六五”普法。启动省市共建食品药品安全示范区，强化安全生产监管，安全生产形势持续趋好。推进韶关矿山救援基地建设，率先在全省建成安全生产应急救援指挥平台。市、县两级人民来访接待厅和县镇村三级综治信访维稳中心全面建成启用，人民调解工作不断加强。强化社会治安综合治理，严厉打击刑事犯罪活动，妥善处置突发事件，平安韶关建设取得新进展。

我市在全省欠发达地区率先普及15年基础教育。学前三年幼儿毛入园率达89.5%，义务教育规范化学校覆盖率达70%。建成国家级示范性农民工培训基地。启动省市共建粤北现代技工教育基地，技工教育培训规模水平居全省地级市前列。高等学校全日制在校生达3.3万人，五年增长38.5%。社区卫生服务中心实现全覆盖，公共卫生服务水平和医疗服务能力持续提升。医疗卫生体制改革稳步推进，全面完成基层医疗卫生机构综合改革。群众性体育活动丰富多彩，竞技体育水平明显提高。全国人口计生综合改革示范市工作扎实推进，低生育水平保持稳定。完成第六次全国人口普查工作。《韶关市志（1988—2000）》出版发行。公共文化服务体系建设不断加强，文化惠民工程扎实推进，打造了《中国之路》等一批文艺精品。民族工作成效显著。积极推进民族团结进步，全力加快少数民族聚居区的经济社会发展。乳源瑶族自治县积极探索后发少数民族地区科学发展新路，为全国创造了民族地区科学发展的鲜活经验。宗教、对台、港澳、侨务、统计、审计、物价、档案、供销、新闻、出版、气象、水文、人民防空、防震减灾、广播电视、无线电管理与信息化等工作取得新进步。

六、不断加强依法行政，行政效能有新提高

坚持向市人大及其常委会报告工作、向市政协通报工作情况，依照法定程序、权限行使行政权力。自觉接受监督，支持人大代表和政协委员履行职责。五年共实施人大议案2件，办理代表建议503件、政协建议案17件和委员提案657件，办复率和办结率均为100%，满意和基本满意率达99.8%。实行重大决策专家咨询论证和听证、公示等制度。完成新一轮政府机构改革和第五批行政审批事项清理。建成全市统一的电子政务基础平台和应用系统。全面落实廉政建设责任制，强化效能监察。严肃查处违法违纪行为，推进网络问政工作，解决了一批关系群众切身利益的问题。

各位代表：过去五年，全市经济持续平稳较快发展，增长速度连续五年高于全国全省水平，固定资产投资、财政收入、金融机构存贷款余额等重要指标实现翻番或接近翻番，“双转移”工作和县域经济发展打开了新局面；城乡人均居民收入和在岗职工年均工资持续较快增长，社会保障制度和体系建设不断加快；交通枢纽初步建成，水利建设取得重大成就；我市成功创建全国双拥模范城、国家卫生城市和国家园林城市，获得“全国文明城市”提名资格；我市承办2011广东国际旅游文化节主会场等一系列重大活动取得圆满成功，先后与澳大利亚宝活市、韩国荣州市、美国旧金山市结为国际友好城市，各级、各单位很多获得了国家和省部级表彰荣誉，扩大了韶关在国内外的影响。这些成绩来之不易，是全市人民团结奋斗的结果。在此，我代表市人民政府，向全市工人、农民、知识分子和干部，向驻韶人民解放军和武警官兵、驻韶中省单位，向各民主党派、人民团体和各界人士，向所有关心支持和参与韶关发展的海内外乡亲和朋友，表示衷心的感谢！

回顾过去五年的工作，我们深刻体会到：要加快发展、缩小差距，必须坚定不移地贯彻落实科学发展观，坚定不移地以经济建设为中心，坚定不移地深化改革、扩大开放，坚定不移地走新型工业化和生态文明发展道路，牢固树立“工业立市、工业强市”理念，紧密团结和依靠全市人民及社会各界，积极争取各方支持，形成推动发展的强大合力。这是全市广大干部群众创造的宝贵经验和智慧结晶，在今后实践中应继续坚持并不断丰富和发展。

同时，我们清醒地看到，我市经济发展不够快，产业规模不够大，加快新型工业化、实现跨越发展还要艰苦努力；城市辐射带动力不强，城乡差距仍然较大，提高城市化质量、促进城乡一体化发展还要艰苦努力；社会建设和管理创新力度不够大，基本公共服务不够完善，一些城乡居民生活仍较困难，改善民生、促进社会和谐稳定还要不懈努力；制约科学发展的陈旧观念和体制机制障碍仍有不少，干部作风有待进一步转变，深化改革、优化软环境还要不懈努力。对此，要高度重视并认真解决。

新一届政府的目标任务和2012年工作意见

今后五年，是我市实现跨越发展、建设幸福美好韶关的关键时期。我们面临许多机遇，也面临不少挑战，机遇大于挑战。我们要认真贯彻落实市第十一次党代会精神，坚持绿色发展，振兴工业经济，加快建设粤北区域中心城市，大力实施“绿色发展、工业强市、文化名城、人才优先、城乡统筹”五大战略，切实增强加快发展的危机感、责任感和紧迫感，抢抓机遇，实干巧干，大力建设实力韶关、活力韶关、宜居韶关、文明韶关、法治韶关、和谐韶关，推动经济社会科学发展，确保实现“十二五”规划目标。

——加快新型工业化步伐，增强产业实力，推动经济跨越发展。依靠加快科技进步、承接产业转移和促进信息化工业化深度融合，改造提升发展支柱产业，推动战略性新兴产业上规模，力争承接产业转移在全省重点区域领先，建成若干年产值超100亿元的特色园区，打造广东装备制造业总部基地、新型干法水泥基地、清洁能源基地、生物制药产业基地和现代中药产业基地取得突破。基本建成优质农产品生产加工基地，现代农业形成规模。发挥区位交通优势，初步建成现代商贸物流基地。推动旅游业率先跨越发展，建设省级旅游产业集聚区，初步建成全国生态旅游休闲重点地区。

——加快改革开放步伐，健全体制机制，增强发展活力。突出抓好大力培育发展和规范管理社会组织、深化住房保障制度改革和医疗保障制度改革三大重点改革任务，推进国有企业及财政金融体制、投融资体制、行政管理体制、社会管理体制、文化体制、人才体制和农村综合改革，不断增强推动跨越发展的内生动力。深入开展全民创业，努力实现民营经济跨越发展。扩大对外开放，深化区域合作，争取引进外商直接投资、外贸出口总额翻一番，引进一批跨国公司和中国500强企业在韶设立生产基地、研发中心和区域总部。

——加快建设中心城市，统筹城乡发展，提升宜居水平。强化规划引领和控制，全面推进芙蓉新城和莞韶产业园工业新城开发建设，加快老城区改造提升，加速马坝组团融入中心城区，增强粤北区域中心城市的交通枢纽、产业集聚、商贸物流、旅游休闲、医疗服务、文化教育等功能，力争粤北山区及粤湘赣相邻地区中心城市建设取得重要进展，韶关都市区初步成形。完善县（市）城区和中心镇基础设施，统筹建设美丽乡村。加强节能减排和自然生态建设保护，加快建设全国生态文明建设示范市，力争生态环境质量在全省和周边地区领先。

——加快建设文化名城，充分发挥社会主义核心价值导向引领作用，推动城乡文明进步。提升全民思想道德素质和科学文化素质，加强文化基础设施建设，发挥文化资源优势，创新文化体制，繁荣文化事业，壮大文化产业，推进文化惠民工程，力争跻身全国文明城市和国家历史文化名城行列，具有韶关特色的文化产业取得新发展。

——加快转变政府职能，提升行政效能，打造良好软环境。坚持依法行政、科学施政、从严治政，加快转变职能，创新管理方式，改进工作作风，努力形成行为规范、运转协调、公正透明、廉洁高效的行政管理体制。强化经济调节、市场监管、社会管理和公共服务职能，建设法治政府，提高政府服务竞争力，力争软环境水平居粤北山区和周边地区前列。

——加强社会建设，创新社会管理，着力改善民生，促进社会和谐。扎实推进基本公共服务均等化，全面发展社会事业，提高就业质量，增加城乡居民收入。基本完善覆盖城乡的社会保障体系和基本公共服务体系，创新社会管理，强化安全监管，完善应急管理体系，提高防灾减灾能力，推进平安韶关建设，促进社会和谐，维护社会稳定，不断提高人民群众幸福指数。

今年是新一届市政府的履新之年，也是实施“十二五”规划的关键之年，做好各项工作意义重大。必须认真贯彻落实党的十七届六中全会、中央经济工作会议、省委十届十一次全会和去年12月市委工作会议精神，继续抓住科学发展这个主题和加快转变经济发展方式这条主线，加快转型升级步伐，调整经济结构，主攻工业发展和城市建设，主抓招商引资和实体经济，着力保障和改善民生，进一步扩大内需，增强发展的平衡性、协调性和可持续性，保持经济平稳较快增长，开创经济社会又好又快发展新局面。

2012年全市经济社会发展主要预期目标是：生产总值增长12%以上，固定资产投资增长16%以上，社会消费品零售总额增长16%，实际利用外资增长12%，地方财政一般预算收入增长10%以上，城镇居民人均可支配收入增长8.5%以上，农村居民人均纯收入增长8.5%以上；市区居民消费价格指数涨幅控制在4%左右，城镇登记失业率控制在3%以内，人口自然增长率控制在7.5‰以下；完成省下达的节能减排任务。围绕实现以上目标，突出抓好以下九个方面的工作：

一、全力以赴实施工业强市战略，扩大承接产业转移规模

牢固树立“工业立市、工业强市”理念，完善支柱工业发展规划并加大组织实施力度，推动工业快速发展，加快经济结构调整和新型工业化步伐。大力发展装备制造、钢铁、有色金属、烟草、电力、制药、玩具等七大支柱工业，着力把新型建材、精细化工、食品饮料及农产品加工业等培育成新的支柱工业。加快发展新兴产业，大力发展高端电子信息、生物医药、新能源、新材料等高科技产业。以生产终端产品、整机产品的大企业为龙头，大力延伸、补足、构建产业链，走产业集群化发展道路。支持企业建立发展战略联盟，强化协作配套，实现共赢发展。突出抓液压油缸、汽车零部件、大型铸锻件、整机装备和成套设备产业发展，加快推进比亚迪汽车零部件和东阳光铝箔增资扩产项目建设，扶持宏大齿轮发展重型卡车变速箱，加快打造省市共建装备基础零部件先进制造业基地及汽车零部件基地。积极申报建设国家级钢材、铅锌产品质检中心等公共技术服务平台，推动丹冶扩建和韶冶异地搬迁改造，延伸烟草工业产业链。推进韶关电厂上大压小、华电热电冷联供、国电煤矸石发电、国电风电等项目，积极开发利用太阳能等可再生能源，加快打造广东清洁能源基地。加快丹霞制药、东阳光药业项目建设和重点制药企业改造，推动制药产业升级。支持旭日、万达、建溢等企业发展壮大，打造韶关玩具品牌。推动台泥、鸿发、南方水泥等大水泥项目和建筑陶瓷项目加快实施，促进精细化工、涂料产业加快集聚，支持青岛啤酒公司、统一集团在我市发展啤酒和矿物质水产业。

加快推进国企改革，健全国有资产监管运营体系，确保国有资产保值增值。积极引进战略投资者，有效盘活存量资产。加快建立健全促进实体经济发展的政策体系、制度体系和服务体系，建立企业发展成长机制和维护机制，促进企业实体数量较快增长。强化煤电水油运和土地等要素保障，实行分类调控，支持优质企业增产增效、加快发展。创新投入方式，充分发挥财政扶持资金作用，撬动信贷资金、民间资金加倍投入中小企业。强化支持中小企业的政策措施，加快中小企业创业载体建设，促进民营经济持续快速发展。鼓励企业实施名牌战略，全面推进质量强市工作。

加快承接产业转移，开辟产业升级新途径。坚持以“大招商、招大商”统领经济工作，走质量招商之路。完善招商引资工作机制和政策体系，重奖做出突出贡献的人员，调动各级干部、社会各界积极性，扎实推进产业链招商及重点产业招商，加快发展总部经济。强化签约项目落地工作，促进已落户项目加快建设。抢抓省加大力度扶持重点区域、重点园区、重点产业的机遇，推进园区扩园扩权扩产，构建高效的园区管理体制和运行机制。支持莞韶产业园以打造广东装备制造业总部基地为战略目标，扩大经济管理权限，加快园区开发建设，扩大招商引资成果，完善配套设施，打造工业新城。加快韶关高新区及高新技术产业发展，加强创新团队和公共创新平台建设，推动科技成果转化和产业化实现新突破。积极运用高新技术和信息技术改造提升优势传统产业，实施数字化改造工程，加快优势企业产品结构优化调整，增强核心竞争力，培育更多企业进入省“优势传统产业100强”和“先进制造业100强”，推进现代产业体系建设。大力推进和扶持县域经济发展，加快培育县域特色工业，促进县（市、区）经济错位发展、合作共赢，支持乐昌纺织服装及钟表、南雄精细化工、仁化有色金属循环经济、始兴制笔研发制造、翁源华彩涂料、新丰华夏生态建筑陶瓷及稀土高新材料、乳源氯碱化工及东阳光生物制药等特色基地建设发展，支持翁源广业科技孵化园建设。开展用地绩效评价，引导企业节约用地。加强与珠三角、“红三角”、港澳台及国际友好城市的交流合作，积极主动与长三角及中西部地区开展经贸合作。加快转变外贸发展方式，促进加工贸易转型升级。

二、千方百计扩大投资规模，加快基础设施和中心城市建设

实行市领导和部门联系跟踪重点项目制度，加快在建项目实施进度，加强交通、能源、水利、信息网络等基础设施建设，加大产业项目投资力度，力争全年固定资产投资超过550亿元，工业投资比重明显提高。加强重大项目策划、包装、储备和报批工作，争取更多项目纳入国家和省的计划。加快赣韶铁路、广乐高速公路（韶关段）、大广高速（新丰段）建设，力争昆汕高速（翁源段）、粤湘高速（韶关段）、韶贺铁路、韶关机场和北江航道“五改三”等项目早日动工建设，进一步完善全市旅游交通设施。加快推进“百亿水利工程”建设。全面建成乐昌峡水利枢纽工程。加快韶钢节能减排、大宝山矿铜硫采选等项目建设，争取韶关核电、南水引水等重大项目早获批准立项。加强投资引导与服务，提高政府投资项目管理水平。优化金融生态环境，拓宽融资渠道。鼓励金融机构加大信贷投放力度，吸引更多金融机构来我市设立分支机构，构建多元的融资平台，推进资本市场工作，培育支持有条件的企业上市融资。强化促进民间投资政策措施，进一步扩大社会投资。

强化规划引导，统筹推进芙蓉新城建设、工业新城建设、老城区改造提升和马坝组团融入中心城区，推进智慧城市建设，加强城市综合管理。加大力度整治违法用地和违章建筑。编制芙蓉新城控制性详细规划，建设芙蓉隧道和滨江路，推进碧桂园太阳城和恒大项目建设，力争行政第二组团、滨江景观带等项目早日开工。把加快“三旧改造”作为重要工作来抓，推进一批重点改造项目，完成既定任务。加强市区自然景观、人文景观和绿化美化亮化工程建设，提升人居环境质量。推进东环路建设和国道323线五里亭大桥西侧至原二棉厂路段、省道248线百旺大桥东侧至转溪路段改造。加快发展城市公共交通并逐步向镇村延伸。积极解决市区停车难问题，有效缓解交通拥堵。支持条件具备的中心镇加快发展，培育打造一批旅游镇、工业镇和商贸镇。

三、大力发展农业和农村经济，推进社会主义新农村建设

认真贯彻落实中央农村工作会议精神，大力落实强农惠农富农政策。加快农业科技进步，促进农业结构调整和农民收入持续较快增长。大力发展优势主导产业，推进农业标准化和品牌化建设，抓好“米袋子”和“菜篮子”。发展壮大农业龙头企业和农民专业合作组织，促进农业集约经营，向“一地一业”、“一村一品”专业化发展。实施“接二连三”战略，推进专业镇建设和转型升级。加强农业招商引资工作，引进大型农产品生产加工流通企业，加快建设粤北现代农业示范园区、粤台（韶关）农业合作试验区、精品农业示范区和华南大宗农产品物流交易中心，积极创建国家现代农业示范区，努力打造面向珠三角和港澳地区的优质农产品生产供应基地。继续把扶贫工作作为一场硬仗来打，巩固扩大工作成果，建立长效机制，确保扶贫开发“双到”工作任务如期完成。加大对老、少、边、穷地区的扶持力度，推进“两不具备”村庄人口异地搬迁工作。以创建名镇、名村示范村为抓手，全面实施农村新村建设规划，扎实推进新农村建设和村庄整治。扩大公共财政覆盖范围，重点加强农村水、电、路、气、房建设，改善农村教育、文化、卫生、体育等基本公共服务，增强防御气象灾害、地质灾害的能力。帮助95个自然村水库移民改造住房。引导农村人口到城镇居住，有序推进空心村改造和特色村保护工作。

四、着力推动旅游业率先跨越发展，带动服务业全面繁荣

创新旅游发展机制，完善旅游发展规划，加强旅游重大项目和旅游产业集聚区建设。完善旅游基础配套设施，建设旅游集散中心。力争创建国家级5A旅游景区1个，新增国家级4A旅游景区2个，新开工建设五星级标准酒店3家以上。围绕“大丹霞、大南华、大南岭、大珠玑”建设，努力整合全市资源，大力发展会展经济，创建会议之都。加快发展文化娱乐、休闲、探险、户外运动等旅游新业态，推进乡村旅游和星级农家乐建设。创新营销形式，塑造“元起丹霞、禅蕴韶关”旅游城市形象，打造“韶关行、好心情”旅游城市品牌，力争全年接待旅游者超过2千万人次、旅游总收入增长20%以上。大力发展专业市场和限额以上商贸企业。加快粤北国际物流、乐昌粤湘仓储物流中心和金友集团物流仓储基地以及光华机电五金、五洲国际汽配用品商贸城等项目建设，主动对接“广东现代流通大商圈”，建设粤北商贸物流大走廊及商贸物流基地。继续实施“农村消费升级行动计划”和节能产品惠民工程，抓好家电、汽车下乡和农机购置补贴等工作。巩固扩大住房、汽车、通讯、文化教育、体育强身、养生保健等消费热点。促进房地产业平稳健康发展。建立鼓励购买使用本地优质产品、优质服务的机制。进一步规范市场秩序，严厉打击制售假冒伪劣商品违法犯罪活动，保持物价基本稳定，营造诚信经营和放心消费环境。

五、扎实推进生态文明建设，切实提升城乡环境质量

全面落实节能减排目标责任制，通过供电与产出挂钩等手段，确保完成节能减排任务。强化节能、环保、土地、安全等要素约束，以经济杠杆倒逼落后产能加快退出，腾出空间发展先进制造业。加快实施重点节能降耗减排工程，抓好重点耗能企业、用能企业节能改造。继续抓好大气、水体、重金属和农业面源污染防治，强化对重点流域、重点企业的监测。加强工业园、中心镇污水处理设施建设。加快建设粤北危险废物处理处置中心，推进工业固废和医疗废物集中处置。治理规模化畜禽养殖污染。深入开展乡村“清洁美”工程。加强水土保持、中小河流综合治理和矿山地质环境恢复治理。推进节水、节材、节地工作，严格保护耕地。加强矿产资源保护与合理开发，发展资源回收利用产业。大力实施林业生态“九大工程”，推进封山育林、林分改造和生态景观林带建设，创建省级林业生态市。

六、大力保障和改善民生，全面发展社会事业

依法强化税收征管，优化财政支出结构，加强预算管理，厉行勤俭节约。抓住国家批准我市适用资源枯竭型城市优惠政策的机遇，加大民生领域投入。大力发展妇女儿童事业，编制实施我市《妇女儿童发展规划（2011—2020）》。实施更加积极的就业、创业政策，加强职业技能培训，推进创业型城市建设。全年城镇新增就业人数5万人，城镇失业人员再就业4万人，新增转移农村富余劳动力就业8万人。实现城乡居民养老保险制度全覆盖，扩大城乡低保覆盖面。提高企业退休人员基本养老金，提高城乡低保标准，提高农村五保老人供养和优抚对象补助水平。实施城镇无医疗保障老年人和无固定职业人员门诊费用报销政策。完善救灾减灾工作体系，加强扶贫济困，促进社会福利、慈善、养老等事业发展。对80周岁以上高龄困难老人发放津贴。抓好保障性住房投融资、建设、运营、管理工作，推进棚户区改造，完成省政府下达的住房保障目标任务。逐步加大对人才建设的投入力度，支持企事业单位人才引进工作。增加教育投入，加快创建省“教育强市”工作，完善教育体系，促进各类教育协调发展。实施“人才强教”工程，提高教育质量。加快推进省市共建粤北现代技工教育基地。全市义务教育规范化学校覆盖率达到80%以上。深化医药卫生体制改革和公共医疗资源整合，推进区域医疗服务中心建设。稳妥开展公立医院改革试点，巩固基层医疗卫生机构综合改革成果，完善基本医疗卫生制度。全面完成社区卫生机构和乡镇卫生院标准化建设。实施全民健身计划，加快乡镇农民体育健身工程建设。推进全国人口计生综合改革示范市建设，稳定低生育水平，促进人口长期均衡发展。加强国防教育、国防动员、民兵预备役和优抚安置工作，开创双拥共建新局面。办好八件关系民生的实事：1、建设武江河市区饮用水源保护区防护工程；2、在芙蓉新城启动建设市图书馆新馆；3、建设400公里通自然村硬化公路；4、推动新型农村社会养老保险全覆盖；5、建设百芙绿道；6、全面启动原曲仁矿棚户区改造二期项目；7、建设韶关市残疾人康复中心；8、建设市区公益性自行车租赁站网。

七、加快推进文化名城建设，促进社会文明进步

坚持以社会主义核心价值体系为引领，广泛开展群众性精神文明创建和群众性文化活动，加强公民道德建设，弘扬优秀传统文化，营造美好精神家园，推进全国文明城市创建，塑造人文韶关新形象。加强文化遗产保护和文化设施建设，推进国家历史文化名城创建。推进市、县（市、区）、镇（乡、街道）、村（居）四级文化设施达标建设并向公众免费开放，继续实施文化信息资源共享等重点文化惠民工程。加强基层文化队伍建设，着力培育文化人才，支持精品创作，打造文艺精品，扩大本地特色客家文化影响力。广泛开展文化活动。深化文化体制改革，培育文化骨干企业。推进大南华文化旅游创意产业项目和乳源世界过山瑶文化项目建设，促进文化与旅游融合，发展特色文化产业。

八、切实加强社会建设，创新社会管理

着力构建政府、社会、公民共同参与的社会治理机制和社会管理新格局。推进社会信用体系建设，加强社会组织、特殊人群和互联网虚拟社会管理。健全社区管理体制，加强流动人口服务和管理。严格落实安全生产责任制，严防事故发生。推进省市共建食品药品安全示范区，提升食品药品安全保障水平。加强校园、校车安全管理，努力保障师生安全。支持县（市、区）建成职工社会化法律维权服务中心。加强法律援助，支持做好司法救助。加强信访和人民调解工作，妥善解决群众合法合理诉求。强化社会治安综合治理，依法打击犯罪活动，努力保障人民生命财产安全，维护社会稳定。继续做好民族、宗教、外事、侨务、新闻、出版、对台、港澳、统计、物价、档案、人防、供销、史志、普法等工作。

九、加强民主法制建设，打造高效廉洁的服务型政府

自觉接受市人大及其常委会的法律监督、工作监督和市政协的民主监督，认真办理人大议案、代表建议和政协提案。认真听取各民主党派、工商联、无党派人士和各人民团体的意见，支持工会、共青团和妇联等人民团体发挥作用。严格按照法定权限和程序行使权力、履行职责。推进山区县综合改革，扩大县（市、区）政府管理权限。改革政府工作流程，提高工作效率。统一安排、集中时间进行检查考核工作，让县级政府有更多时间干实事。积极稳妥推进事业单位改革。结合开展第六轮行政审批事项清理和深化审批制度改革，进一步优化政务环境。扩大政府向社会组织购买服务，制定出台市级第一批政府转移职能目录和购买服务目录。完善充实行政服务中心功能，逐步建设成为综合政务中心。实施电子政务畅通工程，提高公共服务信息化水平。健全会议制度，减少开会时间。完善部门绩效考评方式，突出重点，考核实绩，加强问责。严格落实廉政建设责任制，推进公共资源公开集中交易机制改革和部门干部交流任职，加强和创新预防腐败机制，加大制度防腐力度。加强廉政监察、执法监察、审计监督及重点领域监督，坚决查处和纠正损害群众和企业利益的部门行为和行业不正之风。

各位代表：新一届政府任务艰巨、使命光荣。让我们紧密团结在以胡锦涛同志为总书记的党中央周围，高举中国特色社会主义伟大旗帜，以邓小平理论和“三个代表”重要思想为指导，深入贯彻落实科学发展观，在市委的坚强领导下，进一步解放思想，开拓前进，为建设幸福美好韶关而努力奋斗！
